# Induction of APOBEC3B cytidine deaminase gene in HTLV-1 infected T-cells of ATL model mouse

**DOI:** 10.1186/1742-4690-12-S1-P11

**Published:** 2015-08-28

**Authors:** Masakazu Tanaka, Jinchun Yao, Sung-il Lee, Yihua Ren, Norihiro Takenouchi, Jun-ichi Fujisawa

**Affiliations:** 1 Dept. Microbiology; 2 Institute of Biomedical Science, Kansai Medical University, Hirakata, Osaka, Japan

## 

A variety of mutations are accumulated in the genome of HTLV-1 infected T-cells during ATL development. To elucidate the mechanism of ATL development a mouse model of ATL was established by infecting HTLV-1 to humanized NOG mice and the infected mice recapitulate the ATL-like symptoms and die of leukemia within several months of infection. Analysis of gene expressions in the humanized mouse model of ATL demonstrated the induction of APOBEC3B (A3B) gene in the HTLV-1 infected human T-cells. A3B is a member of the APOBEC family of cellular cytidine deaminase and was recently identified as the mutational source in multiple human cancers. We have previously shown that HTLV-1 infected CD25 (-) CD4 T-cells but not CD25 (+) CD4 T-cells in ATL model mouse express a small amount of Tax mRNA even though both cell populations consist of identical infected-cell clones. As the A3B expression in HTLV-1 infected CD25 (+) T-cells was similar to, or rather higher than that in CD25 (-)T-cells, Tax appears not to be involved in the induction A3B. In contrast, ex vivo culture of infected T-cells, in which Tax expression was highly enhanced in both CD25 (+) and CD25 (-)T-cells, resulted in the suppression of A3B expression. However, ectopic expression of HTLV-1 in Jurkat cell by the transfection of HTLV-1 molecular clone plasmid enhanced the expression of A3B gene. It is thus suggested that HTLV-1 infection but not Tax induces the A3B expression. Interplay between HTLV-1 infection and A3B and the possible involvement of A3B in the mutagenesis of host cell genome are currently investigated.

